# Investigating potential causal relationships between SNPs, DNA methylation and HDL

**DOI:** 10.1186/s12919-018-0117-x

**Published:** 2018-09-17

**Authors:** Lai Jiang, Kaiqiong Zhao, Kathleen Klein, Angelo J. Canty, Karim Oualkacha, Celia M. T. Greenwood

**Affiliations:** 1 Department of Epidemiology, Biostatistics and Occupational Health, McGill University, 1020 Pine Avenue West, Quebec, Montreal H3A 1A2 Canada; 2 Lady Davis Institute for Medical Research, Jewish General Hospital, 3755 Côte Ste. Catherine, Quebec, Montréal H3T 1E2 Canada; 3 Département de mathématiques, Université du Québec à Montréal, 405 Rue Sainte-Catherine Est, Quebec, Montréal H2L 2C4 Canada; 4 Departments of Oncology and Human Genetics, McGill University, 3640 rue University, Quebec, Montreal H3A 0C7 Canada; 5 Department of Mathematics and Statistics, McMaster University, 1280 Main Street West, Hamilton, Ontario L8S 4K1 Canada

## Abstract

Using data on 680 patients from the GAW20 real data set, we conducted Mendelian randomization (MR) studies to explore the causal relationships between methylation levels at selected probes (cytosine-phosphate-guanine sites [CpGs]) and high-density lipoprotein (HDL) changes (Δ*HDL*) using single-nucleotide polymorphisms (SNPs) as instrumental variables. Several methods were used to estimate the causal effects at CpGs of interest on Δ*HDL*, including a newly developed method that we call *constrained instrumental variables* (CIV). CIV performs automatic SNP selection while providing estimates of causal effects adjusted for possible pleiotropy, when the potentially-pleiotropic phenotypes are measured. For CpGs in or near the 10 genes identified as associated with Δ*HDL* using a family-based VC-score test, we compared CIV to Egger regression and the two-stage least squares (TSLS) method. All 3 approaches selected at least 1CpG in 2 genes—*RNMT;C18orf19* and *C6orf141*—as showing a causal relationship with Δ*HDL*.

## Background

Individuals and families in GAW20 data participated in the National Institutes of Health (NIH)-funded Genetics of Lipid Lowering Drugs and Diet Network (GOLDN) study of the effects of lipid-lowering drugs and diet on triglycerides and other atherogenic phenotypes. DNA methylation from the Illumina Infinium 450 K array, triglyceride levels, and high-density lipoprotein (HDL) levels were measured on participants before and after 3 weeks of treatment with micronized fenofibrate. Previous studies have identified strong associations between methylation at 4 cytosine-phosphate-guanine sites (CpGs) within *CPT1A* on chromosome 11 and 2 lipid phenotypes [1], and found genetic variants that demonstrated association with the magnitude of the lipid response to treatment [2].

Statistically significant associations between methylation levels and blood lipids could arise from a causal relationship, such that changes in methylation levels at a particular locus induce changes in blood lipids. However, there are many other reasons why a statistical association might be seen, including the likely possibility of confounding, where a third factor influences both the methylation levels and the blood lipids. In the GOLDN clinical trial, fenofibrate treatment may have induced both changes in methylation levels as well as changes in lipids, in the absence of any direct relationship between these two measures. Our goal was to explore the use of Mendelian randomization (MR) methods, a type of instrumental variable analysis, to try and elucidate the causal relationships between methylation and blood lipids in the GAW20 real data set. This may shed some light on the mechanism of action of the treatment. As there are single-nucleotide polymorphisms (SNPs) with strong associations with CpGs on the Illumina array, these might make good instruments in an MR analysis exploring causality between methylation and lipids, to distinguish between spurious association resulting from confounding and a potentially causal relationship.

Strong SNP associations are needed for a successful MR analysis. Although there are strong associations between pretreatment methylation levels and several genetic variants, we did not see strong associations between changes in methylation levels (pre−/posttreatment) and the SNPs. Consequently, here we have explored the potential causal relationships between *pretreatment* methylation and the HDL treatment response, that is, the changes pre−/posttreatment (Δ*HDL*). Hence, we can be sure that there is no reverse causation (lipid changes cannot alter pretreatment methylation). Another key assumption for MR analysis is that there is no pleiotropy, such that the SNPs are associated with the outcome (Δ*HDL*) only through the intermediate phenotypes (methylation). Here, we also investigate the performance of a new method that tries to account for potential pleiotropy by selecting SNPs with strong associations with the intermediate phenotype of interest, and little association with potential pleiotropic phenotypes.

## Methods

Based on genes identified as associated with Δ*HDL* in family-based variance-component association tests (see Zhao et al. [3]), we selected 10 genes to explore causal relationships. Methylation probe sets were created to include all probes in a window defined by (start − 20 kb, end+ 20 kb) of each gene, to capture probes that could be implicated in *cis*-regulation. To adjust for potential unexplained confounding, principal components (PCs) capturing genome-wide variations in methylation levels were calculated from 2000 randomly sampled probes from all autosomes (see Zhao et al. [3]). The pretreatment methylation levels and Δ*HDL* were then adjusted for the fixed effects of the top 4PCs as well as age, sex, smoking, center, fast time, and metabolic syndrome status, and for a random effect with covariance based on the kinship matrix, to capture effects resulting from familial relationships. Residuals were used for further MR analyses. SNPs were selected in a large window around each gene (start − 400 kb, end+ 400 kb). The large size of these windows was necessary to ensure enough SNPs for the constrained instrumental variables (CIV) method described below. Missing values, approximately 0.5% of all SNP data, were imputed using the K-nearest neighbor method with the Bioconductor package *impute*. When SNPs in the set were highly correlated (*p* > 0.8) with neighboring SNPs, we kept only 1 SNP closest to the 5′ end of each cluster. The resulting SNP set is referred to as the full set of SNPs (or *F*). Univariate linear models were fit between the pretreatment methylation residuals for each CpG near the selected genes, and each retained SNP near the same gene. Based on these linear regression results, reduced sets of SNPs (*R*), with significant F-statistics (*p* < 0.05), were constructed for use with some of the MR methods.

MR analyses using two-stage least squares (TSLS) [4], Egger regression [5], and our new method, CIV, briefly described below, were performed to evaluate the potential causal effects of variability in pretreatment methylation levels (*X*) on Δ*HDL* (*Y*). In TSLS and Egger regression, SNPs (*G*) are used to estimate the exposure $\widehat{X}$, and then the outcome, *Y*, is regressed on the estimated $\widehat{X}$ to estimate the causal effect of *X* on *Y*. Egger regression adjusts for some of the possible pleiotropic effects and also detects small sample bias.

The CIV method is designed to adjust causal effect estimates of *X* on *Y* when potential pleiotropic exposures, *Z*, are measured [6]. Naïve inclusion of genotypes with pleiotropic effects among SNPs to be used as instruments may lead to biased estimation of the causal effect. CIV finds a penalized linear projection orthogonal to *Z* to construct a valid and strong instrumental variable. A constrained optimization approach using smoothed penalty functions forces approximately sparse models. The strength of CIV instruments can be measured with a global F-statistic and the concentration parameter [7]. The latter measures the overall association between *X* and *G*, whereas the former also considers the number of instruments used; if there are many weak instruments, this will be reflected in the F-statistic. F-statistics< 10 are often considered weak instruments. Simulation studies [6] have compared CIV with TSLS, Egger regression, and other popular MR methods under scenarios varying the instruments’ relative strength, validity and pleiotropic directions, and showed that CIV estimates causal effects with little to no bias.

For CIV analysis, the neighborhood around each gene of interest was partitioned into 2 subsets: a set of probes where causal inference is desired ({*X*}: the methylation probe set for each gene) and a set of CpGs whose potential pleiotropic effects are of concern ({*Z*}: methylation probe sets for genes up to 100 kb on either side of the probes in {*X*}). For each CpG in {*X*}, causal inference analysis was performed with CIV, TSLS, and Egger methods. Only the CIV method also used the probe set {*Z*} for analysis. For CIV, the full set (*F*) of SNPs was used for analysis; for Egger and TSLS, both sets *F* and *R* were used as instrumental variables. For all methods, bootstrap confidence intervals, based on 200 bootstrap samples, were constructed for the estimated causal effect of *X* on *Y*.

## Results

**Fig. 1:**
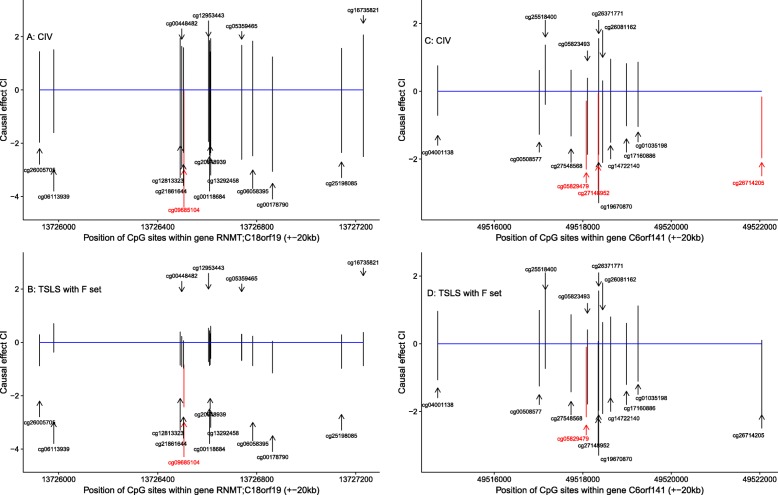
Causal estimates and 95% bootstrap confidence intervals for methylation levels on *ΔHDL* at CpGs in RNMT;C18orf19 (**a**; **b**) and C6orf141 (**c**; **d**), using CIV (**a**; **c**) and TSLS (**b**; **d**) Gene location is indicated in blue. The significant probes identified by CIV/TSLS methods are marked with red

From the results in Zhao et al. [3], we selected the 10 genes with the strongest associations between pretreatment methylation levels and Δ*HDL*. Table 1 shows the number of probes assigned to {*X*} and {*Z*} for each gene, together with the numbers of SNPs in the full and reduced sets, and the number of probes associated with Δ*HDL* in ordinary regressions. After MR analysis, potential causal associations were identified at only 2 of these 10 genes. Figure 1 shows the estimated causal effects for all CpGs at *RNMT;C18orf19* with CIV (Fig. 1a) and TSLS (Fig. 1b), showing that nonzero causal association was estimated at the same CpG, cg09685104, with these methods. In contrast, Egger regression found no associated CpGs at *RNMT;C18orf19*. Even though TSLS and CIV identified the same probe, the SNPs contributing to the instruments differ; Table 2 shows that CIV instrument was much stronger than the TSLS instrument. In fact, for *RNMT;C18orf19*,only CIV constructed a strong instrument (F-statistic> 10). The concentration parameter increases with the number of instruments, and therefore looks smaller for CIV, which implements a sparse solution. For *C6orf141,* there were differences between methods and between SNP sets in which CpGs demonstrated causal associations. CIV (Fig. 1c) suggested that 3 CpGs (including cg05829479) show causal associations; in contrast, TSLS (Fig. 1d) and Egger regression identified the same (and only one) probe, cg05829479, using the reduced set (*R*). At both genes, correlations between CIV instruments and *Z* were minimized (maximum value:1.01e-15) while the raw correlations between *G* and *Z* ranged from 0.1 to 0.5.

**Table 1:** Features of the probes (*X*, *Z*) and SNPs (*G*) analyzed at 10 genes selected due to the associations between methylation levels and Δ*HDL* in Zhao et al. [3]

Gene	CpGs: # in gene ^a^/# nearby ^b^ (# genes nearby)	SNPs: # in Set *F*/# in Set *R*	# probes associated with Δ*HDL* ^c^	Gene	CpGs: # in gene ^a^/# nearby ^b^ (# genes nearby)	SNPs: # in Set *F*/# in Set *R*	#CpGs associated with Δ*HDL* ^c^
*RNMT;C18orf19*	15/31(4)	128/21	1	*GTF2IRD2*	6/20 (4)	0/0	1
*MIR130B*	3/77(9)	90/10	2	*VPS25* ^d^	11/41(19)	59/3	4
*C6orf141*	14/34(5)	53/8	2	*SBSN* ^d^	8/45 (3)	102/4	1
*TUBB3*	20/214 (8)	0/0	1	*TPM4*	29/74 (7)	161/14	1
*TBX15*	109/57(1)	91/63	2	*PARP15*	17/60(5)	146/1	4

^a^ Probes located within 20 kb of the gene of interest

^b^ Probes within 20 kb of neighboring genes, and up to (±100 kb) from the gene of interest; ie, CpGs used as potential pleiotropic phenotypes in the CIV method

^c^ Probes that showed significant associations in univariate analysis at significance level 0.01 with Bonferroni correction

^d^ Only probes within 20 kb (instead of 100 kb) of neighboring genes around genes*VPS25*and *SBSN* were used as *Z*

**Table 2:** MR results with CIV and TSLS at *RNMT*;*C18orf19* and *C6orf141* using the SNP sets *F* and *R*

Gene	# SNPs selected by CIV	Instrument strength of CIV: F-stat/CP ^a^	Instrument strength of TSLS: F-stat/CP ^a^	#Associated probes by CIV ^b^ (# also naïve)	#Associated probes by TSLS ^c^ (# also naïve)
*RNMT;C18orf19*	1	22.83/22.96	(F) 2.21/346.03 (R) 8.88/136.39	1(1)	1(1)
*C6orf141*	11	1.42/27.81	(F) 1.35/78.01 (R) 1.12/23.01	3(1)	1(1)

The number of associated probes based on 95% bootstrap confidence intervals is shown, and the instrument strength at the most strongly associated CpG is also reported. Egger regression with SNP set *F* only identified the probe in *C6orf141*.

^a^ *F-stat*/*CP*,F-statistic/concentration parameter

^b^ Number of CpGs showing significant MR association with CIV (number of these probes also demonstrating naïve association)

^c^ TSLS has the same result using set *F* or set *R*

## Discussion

We compared 3MR methods to look for causal relationships between DNA methylation and Δ*HDL* at 10 genes that showed strong associations. At two of these genes, at least 1 CpG demonstrated evidence of a causal relationship with both TSLS and our new method CIV. The CIV method, as expected, finds stronger genetic instruments (and less correlated with probes in neighboring genes), as this is how it was designed. This does not, however, appear to inflate false-positive findings. Across the 100 simulated GAW20 data sets, we found no causal associations with methylation levels and triglyceride changes at the true CpG sites (results not shown). Furthermore, here we found no causal associations at 8 of the top 10 genes investigated.

One important advantage of the CIV method is that it can automatically select valid instruments from a large candidate SNPset, and does so to maximize instrument strength while minimizing pleiotropic effects. We recognize, however, that there may be potential for overestimation of the strength of the causal relationships using all these MR methods as a result of the relatively small sample size in the GAW20 data. The “winner’s curse”, which leads to biased causal effect estimates, could also happen here since the same data (GAW20) is used for estimating both *G* → *X* and *X* → *Y*.Because CIV contains a penalization step that excludes some SNPs from the instrument, CIV should be less vulnerable to overfitting than methods without penalized SNP selection.

MR analysis with TSLS can be undertaken either with 1 SNP or with a set of SNPs. When SNPs showed weak univariate associations with methylation, TSLS sometimes displayed computational singularities, thereby requiring our reduced set of SNPs. In contrast, CIV requires that the number of instruments be larger than the number of pleiotropic phenotypes, requiring us to use a large window around each gene to capture sufficient SNPs.

GAW20 data does support some causal relationships between pretreatment methylation levels and Δ*HDL*. However, because the selected CpGs have not been previously reported as associated with HDL, further investigation of the adjacent genes and selected CpGs in larger sample sizes may advance understanding of the determinants of HDL treatment responses.

## Conclusions

When using MR to explore causal relationships between pretreatment methylation and Δ*HDL* at 10 genes where they were strongly associated, a new method, CIV, performed automatic instrumental variable selection on a large set of SNPs, constructed genetic instruments accounting for potential pleiotropy, and found stronger instruments than TSLS or Egger regression. Potentially causal relationships were identified at *RNMT;C18orf19* and *C6orf141.*
